# Skeletal muscle in healthy humans exhibits a day-night rhythm in lipid metabolism

**DOI:** 10.1016/j.molmet.2020.100989

**Published:** 2020-04-06

**Authors:** Ntsiki M. Held, Jakob Wefers, Michel van Weeghel, Sabine Daemen, Jan Hansen, Frédéric M. Vaz, Dirk van Moorsel, Matthijs K.C. Hesselink, Riekelt H. Houtkooper, Patrick Schrauwen

**Affiliations:** 1 Laboratory Genetic Metabolic Diseases, Amsterdam UMC, University of Amsterdam, Amsterdam Gastroenterology and Metabolism, Amsterdam Cardiovascular Sciences, Meibergdreef 9, 1105 AZ Amsterdam, the Netherlands; 2 Department of Nutrition and Movement Sciences, NUTRIM School of Nutrition and Translational Research in Metabolism, Maastricht University Medical Center, P.O. Box 616, 6200 MD Maastricht, the Netherlands; 3 Core Facility Metabolomics, Amsterdam UMC, University of Amsterdam, Meibergdreef 9, 1105 AZ Amsterdam, the Netherlands; 4 Diabetes Research Center, Washington University, St. Louis, MO 63110, USA

**Keywords:** Circadian clock, Lipidomics, Lipid metabolism, Human skeletal muscle

## Abstract

**Objective:**

Human energy metabolism is under the regulation of the molecular circadian clock; we recently reported that mitochondrial respiration displays a day-night rhythm under study conditions that are similar to real life. Mitochondria are interconnected with lipid droplets, which are of importance in fuel utilization and play a role in muscle insulin sensitivity. Here, we investigated if skeletal muscle lipid content and composition also display day-night rhythmicity in healthy, lean volunteers.

**Methods:**

Skeletal muscle biopsies were obtained from 12 healthy lean male volunteers every 5 h over a 24 h period. Volunteers were provided with standardized meals, and biopsies were taken 4.5 h after each last meal. Lipid droplet size and number were investigated by confocal microscopy. Additionally, the muscle lipidome was assessed using UPLC/HRMS-based semi-targeted lipidomics.

**Results:**

Confocal microscopy revealed diurnal differences in intramyocellular lipid content (*P* < 0.05) and lipid droplet size in oxidative type 1 muscle fibers (*P* < 0.01). Lipidomics analysis revealed that 13% of all detected lipids displayed significant day-night rhythmicity. The most rhythmic lipid species were glycerophospholipids and diacylglycerols (DAG), with the latter being the largest fraction (>50% of all rhythmic species). DAG levels showed a day-night pattern with a trough at 1 PM and a peak at 4 AM.

**Conclusions:**

Using two distinct methods, our findings show that myocellular lipid content and whole muscle lipid composition vary across the day-night cycle under normal living conditions. In particular, day-night rhythmicity was present in over half of the DAG lipid species. Future studies are needed to investigate whether rhythmicity in DAG is functionally related to insulin sensitivity and how this might be altered in prediabetes.

## Highlights

• Human muscle lipid droplet content varies across the day-night cycle.
• Whole muscle lipid composition varies across the day-night cycle.
• Rhythmicity of the lipidome is associated with fatty acid acyl chain length.

## 1 Introduction

Skeletal muscle contains lipids that are stored in the form of droplets, which fuel mitochondrial respiration. Accordingly, endurance athletes have high amounts of intramyocellular lipids (IMCLs) to support endurance exercise [1]. However, IMCLs are also increased in individuals with low insulin sensitivity where certain bioactive lipids, such as diacylglycerol (DAG) or sphingolipids (SLs), might impede insulin signaling in skeletal muscle [2]. Besides the type of fat, also the characteristics of the lipid droplet itself have been implicated in the etiology of type 2 diabetes, with cells of patients with type 2 diabetes mellitus (T2DM) being characterized by larger lipid droplets—mainly in type 2 muscle fibers—when compared to insulin-sensitive endurance-trained athletes who are characterized by more but smaller lipid droplet [3]. These data indicate that both lipid droplet characteristics and muscle lipid composition are involved in muscle insulin resistance in humans.

In recent years, it has become apparent that metabolic processes in skeletal muscle are regulated by the circadian timing system, presumably to anticipate cycles of activity and food intake during the day [4]. The circadian clock is a transcriptional-translational feedback loop, consisting of the transcription factors BMAL1 and CLOCK (positive limb) and PER2 and CRY1 (negative limb). Over approximately 24 h, this feedback loop generates the autonomous expression of large parts of the genome. Studies in which one of the key regulatory genes of the muscle clock is manipulated show that mitochondrial oxidative capacity and lipid metabolism are affected, indicating a tight connection to the circadian clock [5,6]. Moreover, we recently found that mitochondrial respiration displays a day-night rhythm in healthy, lean individuals, with a peak in mitochondrial respiration that occurs at 11 PM, suggesting that the highest capacity to generate ATP through oxidative metabolism is at the end of the day [7]. At the same time, mitochondrial proteins that are involved in fusion and fission processes (OPA-1 and FIS-1) displayed a day-night rhythm suggesting mitochondrial dynamics as a putative underlying mechanism for rhythmic respiration.

In skeletal muscle, lipid droplets reside in close proximity to mitochondria, hence facilitating the swift delivery of fatty acids for oxidation to the mitochondria [8]. Therefore, it is tempting to speculate that, besides mitochondria, also lipid droplet characteristics and lipid composition are under the control of the circadian clock. Indeed, using lipidomic approaches in human plasma, it has been found that ∼13% of plasma lipid species are rhythmic [9]. In human skeletal muscle, however, so far only one study has investigated circadian rhythmicity of lipids [10]. Using a highly controlled protocol in which volunteers received small hourly meals and were confined to bed rest in order to exclude the acute influence of behavior, more than 20% variation in lipid species abundance over the day was found. Although such a highly controlled approach provides important insight into the role of the circadian clock on muscle metabolism, it can be less easily translated to the potential physiological impact that such rhythms may have on variation in muscle homeostasis, such as insulin sensitivity. In that respect, it is interesting to note that muscle insulin sensitivity is higher in the morning compared to the evening. Whether fluctuations in lipid species and lipid droplet morphology are also seen under free-living conditions is unknown.

In the present study, we analyzed muscle biopsy samples obtained over 24 h using both confocal microscopy for lipid droplet number and morphology and a semi-targeted ultrahigh-performance liquid chromatography-high resolution mass spectrometry- (UPLC/HRMS-) based lipidomic analysis to comprehensively assess skeletal muscle lipids. We obtained muscle samples every 5 h throughout a 24 h period, while subjects remained under controlled laboratory conditions that mimicked real-life conditions with three meals over the day and structured physical activity to assess muscle lipid metabolism under realistic diurnal conditions.

## 2 Material and methods

### 2.1 Participants

Twelve young, lean male volunteers (age ± SD: 22.2 ± 2.3 years, BMI ± SD: 22.4 ± 2.0 kg/m^2^) were recruited for this study. Only participants who slept on average 7–9 h per night and who did not perform shift work or traveled across more than one time zone for at least 3 months prior to the study were eligible to participate. Extreme morning or evening types were excluded based on the morningness-eveningness questionnaire self-assessment (MEQ-SA) version. All participants provided written informed consent. The study was in agreement with the principles of the declaration of Helsinki and approved by the Ethics Committee of the Maastricht University Medical Center. The study was registered at clinicaltrials.gov with identifier NCT02261168.

### 2.2 Study design

Study procedures have been described in detail before [7]. Briefly, one week prior to the study, participants were instructed to maintain a standardized lifestyle (preweek). This lifestyle included bed rest every night from 11 PM until 7 AM, eating breakfast, lunch, and dinner at regular times (at 9 AM, 2 PM, and 7 PM) with no in-between snacks or drinks other than water. Participants were not allowed to drink caffeine and alcohol this week. Lifestyle was monitored by accelerometry (activPAL3 physical activity monitor, PAL Technologies, Glasgow, UK) together with food and sleep diaries. Two days before the study, meals were provided to ensure standardized caloric and macronutrient intake for all participants.

During the laboratory visit, subjects stayed for 44 h inside a metabolic research ward (Supplemental Figure 1). Living conditions were standardized but resembled a real-life situation with regular meals and activity during the day. Meals were provided at fixed times (9 AM, 2 PM, and 7 PM). One hour after each meal, participants went for a 15-minute supervised stroll, followed by 15 min of standing to circumvent complete sedentarism which commonly accompanied metabolic ward studies. Five skeletal muscle biopsies were obtained from the vastus lateralis muscle according to the Bergström method [11] under local anesthesia (1% lidocaine, without epinephrine). Biopsies were taken from separate incisions, which were made a few centimeters apart. In addition, muscle biopsies were taken alternating from the right or left leg. The site (left or right leg) of the first biopsy was randomized. Muscle tissue was immediately frozen in melting isopentane and stored at −80 °C for histochemical analysis.

### 2.3 Study meals

All study meals were standardized and were according to the Dutch and US dietary guidelines. Caloric intake during the preweek was calculated using the Harris-Benedict formula [12] with an activity factor of 1.5 and optional snacks up to an activity factor of 1.7. On study-day one, the activity factor was lowered to 1.35 to account for reduced physical activity in the metabolic ward. On study-day two, sleeping metabolic rate (measured with whole-room indirect calorimetry) was multiplied with an activity factor of 1.5. Participants were provided with three meals each day. Breakfast was ∼21 energy%, lunch ∼30 energy%, and dinner ∼49 energy%. Daily macronutrient composition was ∼52 energy% as carbohydrates, ∼31 energy% as fat (of which 9 energy% saturated), and ∼14 energy% as protein. No snacks and only water to drink were allowed between meals.

### 2.4 Histochemical analyses

Muscle tissue was cut in 7 μm thick sections and mounted on glass slides. To minimize staining intensity variability between subjects, a section from each biopsy from the same participant was mounted on the same glass slide. Cryosections were fixed with 3.7% formaldehyde for 30 min. After blocking with blocking buffer (150 mM NaCl, 20 mM Tris pH 6.8, and 2% BSA) and permeabilization for 5 min with 0.25% TX-100 (648466, Merck, Darmstadt, Germany), sections were incubated with primary antibodies against Laminin (L9393; Sigma) and myosin heavy chain type I (A4.840; Developmental Studies Hybridoma Bank, Iowa City, Iowa, USA) for 60 min. Subsequently, sections were incubated for 1.5 h with the appropriate secondary antibodies conjugated with AlexaFluor 405 and AlexaFluor 555 and Bodipy 493/503 (D3922, Invitrogen-Thermo Fisher Scientific, The Netherlands) 1:100 at 37 °C. Sections were mounted with Mowiol, covered with #1 coverslips, and stored in the dark until imaging. Type I fibers were identified based on positive myosin heavy chain type I staining, and all other fibers were considered to be type II fibers. The fiber type ratio was determined using a Nikon E800 fluorescence microscope prior to confocal imaging to account for bias by differences in fiber type ratio. Cross sections of 20 fibers per participant were imaged with the confocal microscope. After image acquisition, images were deconvolved using Huygens Professional software (Scientific Volume Imaging B.V., Hilversum, The Netherlands). For each fiber type, lipid area fraction, LD size, and number were analyzed by using ImageJ [13]. LD size and distribution were calculated using a customized script for MATLAB R2012a (The Mathworks, Inc., Natick, Massachusetts, USA).

### 2.5 Image quantification

Cross-sectional images were taken on a Leica TCS SP8 STED microscope in confocal mode using a 63 × 1.3 N.A. oil immersion objective and 1.1 optical zoom using 2048 by 2048 pixels, resulting in a pixel size of 81.95 nm by 81.95 nm. Laminin, myosin heavy chain type I and Bodipy 493/503 were imaged using the 405 nm, 532 nm, and 488 nm laser lines, respectively.

### 2.6 Lipid extraction

Muscle biopsies were freeze-dried and powdered to allow equal portioning. 1 mg of dry tissue was suspended in 300 μL MQ water and sonicated on ice for 30 s at 8 W using a tip sonicator. Lipids were extracted using a single-phase methanol-chloroform extraction. The following internal standards (all lipid standards from Avanti Polar Lipids, Alabaster, AL, USA) were added: 0.5 nmol of Diacylglycerol DAG(14:0)_2_; 0.5 nmol of Triacylglycerol TAG(14:0)_2_; 2.5 nmol of Cholesteryl ester CE(16:0); 0.1 nmol of Cardiolipin CL(14:0)_4_; 0.2 nmol of Bis(monoacylglycero)phosphate BMP(14:0)_2_; 2.0 nmol of Phosphatidylcholine PC(14:0)_2_; 0.1 nmol of Phosphatidylglycerol PG(14:0)_2_; 0.5 nmol of Phosphatidylserine PS(14:0)_2_; 1.0 nmol of Phosphatidylethanolamine PE(14:0)_2_; 0.2 nmol of Phosphatidic acid PA(14:0)_2_; 0.5 nmol of Phosphatidylinositol PI(8:0)_2_; 0.625 nmol of Ceramide phosphocholines SM(d18:1/12:0); 0.02 nmol of Lysophosphatidylglycerol LPG(14:0); 0.1 nmol of Lysophosphatidylethanolamine LPE(14:0); 0.2 nmol of Lysophosphatidylcholine LPC(14:0); 0.05 nmol of Lysophosphatidic acid LPA(14:0); 0.125 nmol of Sphingosine SPH(d17:1), Sphingosine SPH(d17:0), Sphinganine 1-phosphate S1P(d17:1), Sphinganine 1-phosphate S1P(d17:0), Lactose Ceramide LacCer(d18:1/12:0), Glucose Ceramide GlcCer(d18:1/12:0), Ceramide-1-phosphate C1P(d18:1/12:0), Ceramide Cer(d18:1/12:0), and Ceramide Cer(d18:1/25:0) dissolved in 120 μL of chloroform/methanol (1:1, v/v)). The internal standard mix and 1.5 mL of chloroform/methanol (1:1, v/v) were added to the muscle homogenates. Subsequently, the mixture was sonicated in a water bath for 5 min, followed by centrifugation at 4^ᵒ^C (16000×*g* for 5 min). The liquid phase was transferred to a glass vial and evaporated under a stream of nitrogen at 60^ᵒ^C. Subsequently, the residue was dissolved in 150 μL of chloroform/methanol (9:1, v/v), and 2 μL for normal phase and 5 μL for reverse phase separation of the solution were injected into the UPLC-HRMS system.

### 2.7 UPLC-HRMS

Lipidomics analysis was performed as described previously with minor changes described in this section [14]. The UPLC system consisted of an Ultimate 3000 binary HPLC pump, a vacuum degasser, a column temperature controller, and an autosampler (Thermo Fisher Scientific, Waltham, MA, USA). For normal phase separation of lipids, 2 μL lipid extract was injected on a LiChroCART 250–4 LiChrospher® Si 60 (5 μm) (Merck) maintained at 25 °C. For lipid separation, a linear gradient consisting of solution A (methanol/water, 85:15, *v*/v) and solution B (chloroform/methanol, 97:3, v/v) was used. Solutions A and B contained 5 and 0.2 mL of 25% (*v*/v) aqueous ammonia per liter of eluent, respectively. The gradient (0.3 mL/min) was as follows: T = 0–1 min: 10%A; T = 1–4 min: 10%A–20%A; T = 4–12 min: 20%A–85%A; T = 12–12.1 min: 85%A–100%A; T = 12.1–14.0 min: 100%A; T = 14–14.1 min: 100%A–10%A; and T = 14.1–15 min: 10%A. For reverse-phase separation of lipids, 5 μL lipid extract was injected onto an ACQUITY UPLC HSS T3, 1.8 μm particle diameter (Waters) maintained at 60 °C. For lipid separation, a linear gradient consisting of solution A (methanol/water, 40:60, *v*/v) and solution B (methanol/isopropanol, 10:90, v/v) was used. Both solutions A and B contained 0.1% formic acid and 10 mM ammonia. The gradient (0.4 mL/min) was as follows: T = 0–1 min: 100%A; T = 1–16 min: 80%A; T = 16–20 min: 0%A; T = 20–20.1 min: 0%A; and T = 20.1–21.0 min: 100%A. A Q Exactive Plus Orbitrap Mass Spectrometer (Thermo Fisher Scientific) was used in the negative and positive electrospray ionization modes. Nitrogen was used as the nebulizing gas. The spray voltage used was 2500 V, and the capillary temperature was 256^ᵒ^C. S-lens RF level was 50; auxiliary gas, 11; auxiliary gas temperature, 300 °C, sheath gas, 48 au; and sweep cone gas, 2 au. Mass spectra of lipids were acquired in negative and positive scan modes by continuous scanning from *m*/*z* 150 to *m*/*z* 2000 with a resolution of 280,000 full width at half maximum (FWHM) and processed using an in-house developed metabolomics pipeline written in the R programming language (http://www.r-project.org [15]). The identified peaks were normalized to the intensity of the internal standard for each lipid class. The concentration of each added internal was previously optimized for muscle tissue. Lipids with a relative abundance of less than 0.05 were excluded from further analyses.

### 2.8 Statistical analysis

Data are presented as mean ± SEM (standard error of the mean). Statistical analysis of lipid droplet rhythmicity was performed using the IBM Statistical Package for Social Sciences, version 25 (SPSS Inc.). The effect of time on the outcome variables was analyzed with linear mixed models. In the case of a significant time effect for variables of lipid droplets, rhythmicity analysis was performed using JTK_Cycle [16]. The rhythmicity of individual lipid species from the lipidomics measurements was performed using JTK_Cycle. The period length for rhythmicity analyses of lipid droplets and lipidomics was set to 20–28 h, and an adjusted *P* value ≤ 0.05 was considered significant. Before performing JTK_Cycle analysis, each time point was divided by the mean of all 5 time points per subject to decrease the intersubject variability. JTK_Cycle analysis and plotting of heatmaps were done in R. Statistical significance was defined as a *P* value < 0.05.

## 3 Results

### 3.1 Lipid droplet morphology shows variations over 24 h

**Figure 1:**
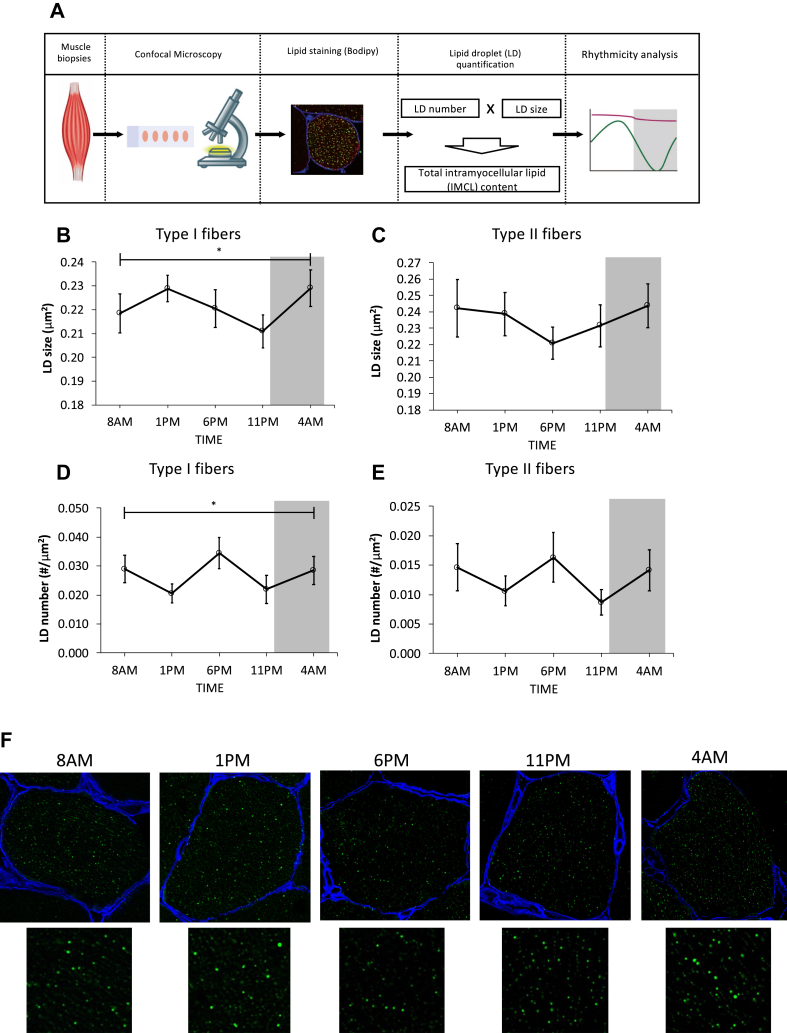
Lipid droplet morphology shows diurnal variation. (A) Workflow for lipid droplet analysis using lipid staining and confocal microscopy. (B) Predominantly oxidative type I fiber lipid droplet size and (D) number show day-night rhythmicity. (C) Predominantly glycolytic type II fibers show diurnal variations in lipid droplet size and (E) number that are not significant. Lipid droplets (green) and cell membranes (blue) were stained and quantified. (F) Representative images of type I fiber lipid droplet size of one subject for the different time points are depicted. Grey area represents sleeping periods (11 PM–7 AM). ∗*P* ≤ 0.05 for the effect of time.

In skeletal muscle, mitochondria and lipid droplets are interconnected, permitting lipid droplets to fuel mitochondrial fat oxidation. To establish if the day-night rhythmicity in muscle mitochondrial function is paralleled by rhythmicity in lipid droplet characteristics, we first examined lipid droplet size and number variation over a normal day-night cycle (Figure 1A). Lipid droplet characteristics have been reported to differ between fiber types [3]. We, therefore investigated lipid droplets separately in type I and type II fibers. Indeed, on average, type I fibers had more lipid droplets, but they did not differ in size (lipid droplet number: 0.027 ± 0.003 versus 0.013 ± 0.003 LD/μm^2^ in type I versus type II, *P* < 0.001; lipid droplet size: 0.221 ± 0.005 versus 0.234 ± 0.011 μm^2^ in type I versus type II, *P* = 0.079). We performed mixed model analysis and found a significant time effect for lipid droplet size in type I fibers, with the largest lipid droplets being towards the middle of the night and the smallest being at the end of the waking period (*P* < 0.001, Figure 1B). The pattern of lipid droplets in type II fibers failed to reach a significant time effect (*P* = 0.416, Figure 1C). However, visual inspection shows that the pattern was different compared to type I fibers. Lipid droplets were the smallest at 6 PM, which is 5 h earlier than in type II fibers. In contrast to lipid droplet size, lipid droplet number did not seem to show day-night rhythmicity but seemed to peak at 8 AM, 6 PM, and 4 AM (Figure 1D,E). We found a significant time effect for lipid droplet number only in type I but not in type II fibers (type I: *P* < 0.001; type II: *P* = 0.416). As a final step, we examined the total IMCL content, computed as the product of lipid droplet size and number in both muscle fiber types. The total IMCL content changed significantly during the day-night cycle and showed a similar pattern as compared to the lipid droplet number (*P* = 0.030 for the effect of time, Supplemental Figure 3A). Despite a significant time effect, rhythmicity quantification using the JTK_CYCLE algorithm did not reveal significant rhythmicity in the total IMCL content (*P* > 0.05 for both fiber types).

### 3.2 Lipidome analysis reveals oscillating lipid species in human muscle

**Figure 2:**
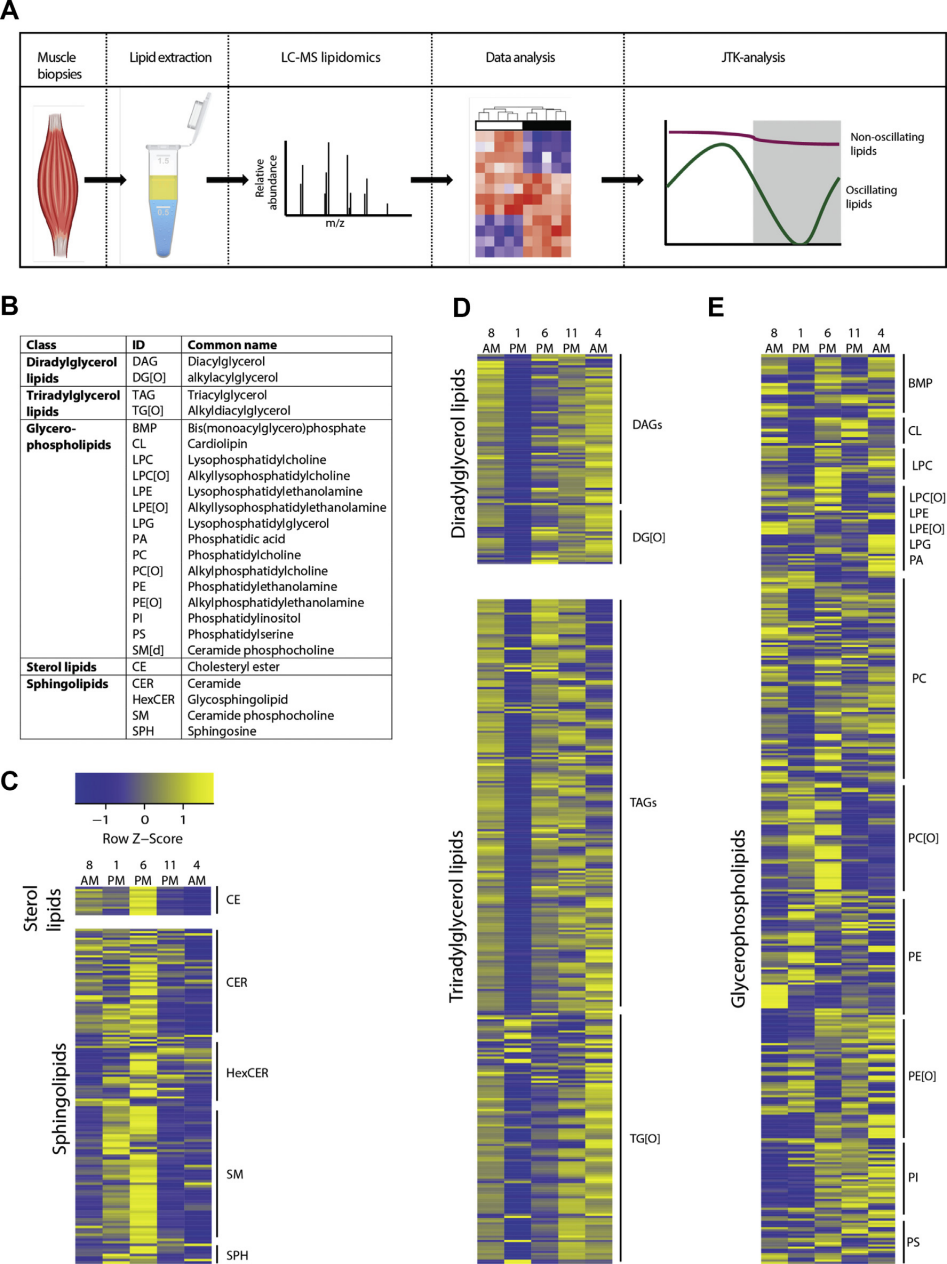
Semi-targeted lipidomic analysis reveals differences in lipid species abundance over 24 h. (A) The workflow of lipidomic analysis using UPLC-HRMS and bioinformatic pipeline. (B) Overview of lipid clusters that are divided into separate lipid classes according to their chemical properties. Heatmaps show z-scores of all detected lipids at 8 AM, 1 PM, 6 PM, 11 PM, and 4 AM. Each time point is the average of all 12 subjects. Lipid species are clustered into (C) sterol lipids and sphingolipids, (D) diradylglycerols and triradylglycerols, and (E) glycerophospholipids.

We next performed targeted lipidomic analysis on all five time points to investigate the temporal changes of individual lipids in the muscle biopsies (Figure 2A). In total, we detected 971 lipid species in 24 lipid classes that were present in all subjects. For a better overview, we summarized the different lipid classes into clusters according to their chemical properties (Figure 2B). This resulted in the following five clusters: (1) diradylglycerol lipids, i.e., diacylglycerols and alkylacylglycerols, (2) triradylglycerol lipids, i.e., triacylglycerols and alkyldiacylglycerols, (3) glycerophospholipids, (4) sterol lipids, and (5) sphingolipids. Through the heatmap analysis, we compared the lipid abundance at all time points for all the detected lipids (Figure 2C and E). The majority of diradylglycerol and triradylglycerol lipids appeared to be the lowest at 1 PM. More specifically, most of the diglycerides (DAGs), longer chain triglycerides (TAGs), and alkylglycerols (i.e., DG[O] and TG[O]) peak at 4 AM (Figure 2D). The cluster of glycerophospholipids was very diverse and contained most lipid species (Figure 2E). Some of these glycerophospholipid classes, such as the phosphatidylcholines (PCs), behaved similarly to the glycerolipids (DAGs and triradylglycerols (TAGs and TG[O])). Overall, the glycerophospholipids appeared to decrease during the beginning of the day and to peak in the evening or early morning. The alkylphosphatidylcholines (PC[O]), however, were the lowest in the evening and early morning. Also, phosphatidylserine (PS) species did not follow the overall trend, as they did not appear to oscillate at all. Sterol lipids (CEs) and sphingolipids (SMs) also did not follow the pattern of glycerolipids, as they peaked at 6 PM and dropped from 11 PM onwards (Figure 2C).

**Figure 3:**
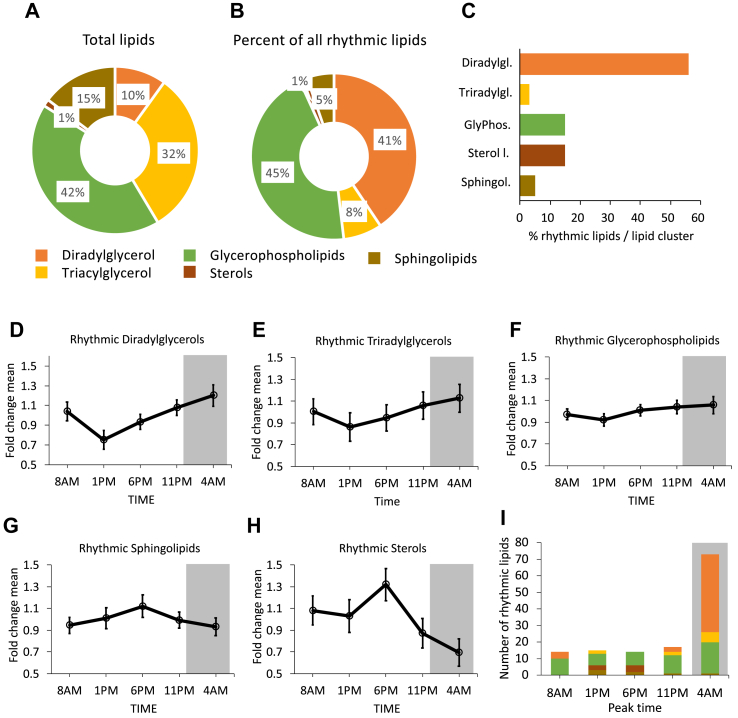
(A) Rhythmic regulation of the skeletal muscle lipidome. Composition of the lipidome (rhythmic and nonrhythmic lipids) according to the five main lipid clusters. (B) Overview of rhythmic lipids and distribution in the main lipid clusters. (C) Bar graph showing the percentage of rhythmic lipids per lipid cluster. More than half of all diradylglycerols are classified as being rhythmic. (D–H) Pattern per lipid cluster shows the average ± SEM per timepoint of all rhythmic lipid species within the cluster. (D) Diradylglycerols, (E) triradylglycerols, (F) glycerophospholipids, (G) sphingolipids, and (H) sterols. (I) Overview of peak times of all lipid clusters. Grey area represents sleeping periods (11 PM–7 AM).

In order to identify lipid species that exhibit statistically significant day-night rhythmicity of ∼24 h, we analyzed all lipids with the JTK_CYCLE algorithm [16]. In total, 126 lipid species were classified as being rhythmic, which was ∼13% of all detected lipids. The distribution of rhythmic lipids did not reflect the total lipid pool. The total lipid pool consisted of mostly glycerophospholipids (∼42%) and triradylglycerol lipids (∼32%) followed by sphingolipids (∼15%), DAGs (∼10%), and sterol lipids (∼1%) of all detected lipids (Figure 3A). The rhythmic lipids, however, were primarily DAGs and glycerophospholipids and only a small percentage of triradylglycerols (Figure 3B). Surprisingly, from the total DAG pool, more than half of them were rhythmic (Figure 3C).

### 3.3 Lipid clusters show distinct patterns in abundance over the day

To better visualize the rhythmicity of the 126 lipid species, we plotted the average of the lipid clusters. The DAGs, triradylglycerols, and glycerophospholipids shared a similar pattern, with a characteristic drop in lipid abundance at noon before increasing again to reach the peak at 4 AM (Figure 3D–F). In contrast, sphingolipids and CEs showed a pattern with a single peak at 6 PM (Figure 3G–H). In order to obtain insight into the heterogeneity within individual lipid classes, we next plotted the individual rhythmic lipid species according to their peak (Figure 3I). This clearly showed that most DAGs (87%) and triradylglycerols (60%) had their peak in the middle of the night (4 AM), whereas most sphingolipids peaked at 1 PM (43%) and most sterol lipids peak at 6 PM (57%). The peaks of rhythmic glycerophospholipid species were much more dispersed across all timepoints.

### 3.4 Chain length and saturation level affect the rhythmic profile of diurnal regulated lipids

**Figure 4:**
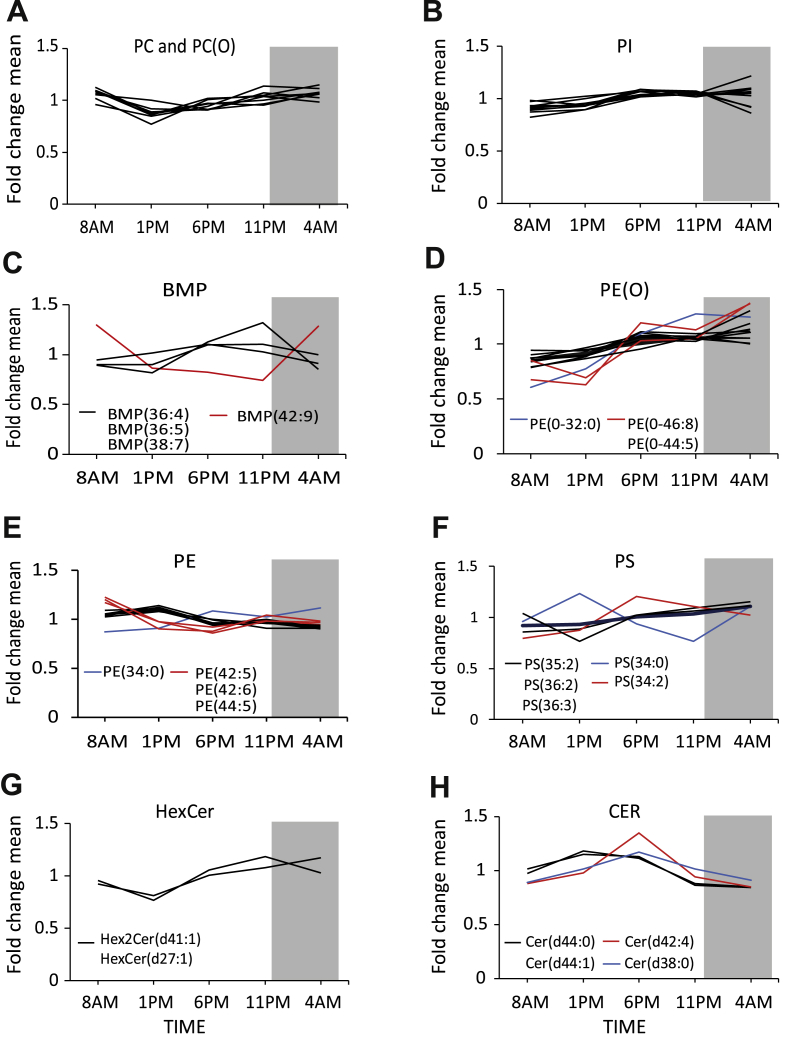
Analysis of individual lipid classes. Rhythmic lipid species in glycerophospholipids in which saturation or chain length has no influence on the pattern such as (A) phosphatidylcholine (PC) and its alkyl-containing counterpart (PC[O]) (B) as well as phosphatidylinositols (PI). Rhythmic lipid species show differences based on saturation or carbon chain length in the subclasses (C) Bis(monoacylglycero)phosphate (BMP), (D) Alkylphosphatidylethanolamine (PE[O]), (E) Phosphatidylethanolamine (PE), and (F). phosphatidylserines (PS). In (G) hexosylceramides (HexCer) and (H) ceramides (CER) showed an opposing rhythmic profile. Black lines depict lipid species that follow a similar pattern within the lipid class. Blue lines depict saturated lipids and red lines depict unsaturated lipids. Grey area represents sleeping periods (11 PM–7 AM).

We next zoomed in on all the individual lipid classes and focused on the rhythmic lipid species. This especially made a difference for the glycerophospholipids as this cluster contained many different lipid classes. Certain rhythmic glycerophospholipid subclasses such as phosphatidylcholine (PC) and its alkyl-containing counterpart (PC[O]) as well as phosphatidylinositols (PIs) showed homogeneous patterns with small differences between the individual lipid species (Figure 4A–B). While other subclasses, such as bis(monoacylglycero)phosphates (BMP), (alkyl-)phosphatidylethanolamines (PE[O], PE), and phosphatidylserines (PS), showed more diversity (Figure 4C–F). The variation in such subclasses appeared to be related to either the carbon chain length of the lipid or the absence (saturated) or the presence of double bonds (unsaturated). Those that consisted of fatty acids with carbon chain lengths longer than 20 carbons often had a different or sometimes even opposing pattern than those consisting of ≤20 carbons. Also, unsaturated lipids appeared to behave distinctly from saturated lipids. For instance, in BMPs, the only lipid species that did not follow the common pattern was BMP(42:9) (Figure 4C). The lipidomic platform we used does not provide the exact fatty acid composition of the lipids but only the total carbon chain length and the total amount of double bonds. Thus, BMP(42:9) could likely be composed of C20:4 and C22:5. All other rhythmic BMPs contained shorter fatty acids such as C16:0, C16:1, C18:1, C:18:2, and C18:3. In PEs, we found a similar trend; those that contained long-chain polyunsaturated fatty acids (LC-PUFAs), such as PE(42:5), PE(42:6), and PE(44:5), showed different rhythmicity compared to those that contained shorter chain fatty acids. Noticeably, PE(34:0) had a distinct pattern and represented the only saturated lipid in the group (Figure 4E). Likewise, the alkylPEs were clustered into three subclusters too, including saturated PE[O](32:0); shorter unsaturated PE[O](30:1), (31:1), (32:1), (32:2), (32:3), (33:1), (34:1), and (37:1); and longer unsaturated PE[O](44:5), (44:7), and (46:8) (Figure 4D).

It is noteworthy that hexosylceramides (HexCer) and ceramides (CERs) showed an opposing rhythmic profile (Figure 4G–H). Interestingly, the expression of the UDP-glucose ceramide glucosyltransferase, an enzyme that catalyzes glycosylation of ceramides, has a similar rhythmic pattern [10]. This may explain why these ceramides have opposing profiles. Fluctuations in sphingolipids and sterol lipids, however, appeared to be independent of chain length or saturation level.

Given that half of all DAGs showed a clear day-night rhythm, we next zoomed in on the differences in the temporal patterns between DAG species and performed clustering, as previously seen in rhythmic glycerophospholipids. The general temporal pattern was not different between short- and long-chain DAGs (Supplemental Figure 2A). Also, the pattern of saturated and unsaturated DAGs was very similar across all time points (Supplemental Figure 2B). However, when the amplitude of all individual DAG species was computed, it appeared that long chain length and more double bonds were associated with larger oscillation amplitude (Supplemental Figures 2C-2D).

## 4 Discussion

We have recently shown that mitochondrial oxidative capacity in skeletal muscle of young, healthy subjects is characterized by a day-night rhythm, with the highest values at the end of the day. Similar rhythms were observed for other markers of muscle- and whole-body metabolism, suggesting comprehensive regulation from the circadian timing system [7]. Here, we report the results of a subsequent in-depth analysis of skeletal muscle lipid metabolism. We show that myocellular lipid droplets are not static but undergo distinct and dynamic morphological changes over the 24 h period under normal living conditions. In addition, we observed 24 h variations of individual lipid species in the majority of lipid classes in the skeletal muscle of healthy volunteers. These data indicate that skeletal muscle lipid metabolism displays 24 h rhythmicity, which potentially could be involved in the 24 h rhythmicity in energy and substrate metabolism.

### 4.1 Diurnal lipid droplet variations

It is well established that the storage of intramyocellular lipids (IMCLs) changes dynamically according to the availability and demand of lipids [17]. For example, lipid infusion [18] or prolonged fasting [19] raises plasma free fatty acids and leads to acute increases in IMCL content. Conversely, acute exercise decreases IMCL depots [20], yet long-term exercise training results in elevated IMCL content [20,21]. However, temporal fluctuations in IMCL content and composition during a normal day with regular meals and normal activity have not yet been explored. One study has investigated changes in IMCLs in the skeletal muscle between morning and evening [22]. In particular, this study used the noninvasive 1H-MRS technique to measure IMCLs in the tibialis anterior muscle and soleus muscle at 8 AM and 6 PM. IMCL levels were not different between morning and evening, which is in line with our findings that total IMCL content was not different between 8 AM and 6 PM (Supplemental Figure 3). However, the pattern of the total IMCL content in our study indicates that IMCL content oscillates more frequently during the day, with more than one peak and trough. IMCL is dispersed within the muscle cell in the form of lipid droplets, which differ in size, number, and location [17] which are in turn associated with cellular function like fat oxidative capacity and insulin sensitivity. The general rhythmic pattern in the lipid droplet number was quite similar between the two fiber types. In both fiber types, the lipid droplet size decreased over the day before reaching a trough in the evening (Figure 1). However, in type II fibers, the peak and trough seemed to occur 5 h earlier than those in type I fibers (at 6 PM versus 11 PM), although the diurnal differences in type II fibers did not reach statistical significance. Regardless of the potential difference between fiber types, the rhythmic pattern in the lipid droplet size indicates a dynamic profile of extraction of fatty acids from lipid droplets throughout the day followed by lipid droplet replenishment during the evening and night, a period when postprandial plasma TAGs gradually start to drop. This may suggest that skeletal muscle helps in the clearance of diet-derived plasma TAGs and that lipid droplets may be refilled in order to provide substrate at other times during the day. In contrast to lipid droplet size, lipid droplet number was on average higher in type I versus type II fibers, which is in agreement with previous studies that compared lipid droplets in insulin-sensitive subjects [3,23]. In addition, the lipid droplet number in both fiber types showed a very similar 24 h rhythmicity. The pattern in the lipid droplet number with peaks in the night/early morning (4 AM/8 AM) and early evening (6 PM) also seemed to contribute most to the overall IMCL pattern (Supplemental Figure 3). Several factors such as feeding/fasting or rest/activity could determine the observed rhythmicity in lipid droplet size and number. Intravenous lipid infusion during hyperinsulinemia leads to a relatively fast increase in the total IMCL after 6 h [18,24]. However, under physiological conditions in which a high-fat meal is consumed, IMCL does not change after 3 h [25]. Thus, it seems unlikely that the rhythm of the lipid droplet size and number is mainly determined by the meal schedule. Similarly, it was found that exercising for 2 [26] or 3 [20] hours at moderate intensity leads to a decrease in the IMCL of 17% or 14%, respectively. This is substantially more physical activity than walking at a low intensity for 15 min one hour after the meal, as the participants did in our study. Disentangling the effects of physiological behaviors, such as diet and physical activity, on IMCL and lipid droplets would require alternative study protocols that do not resemble natural living conditions, such as constant bedrest or small hourly meals [27].

### 4.2 The effect of a 24 h rhythm under free-living conditions on the muscle lipidome

Lipid droplets store neutral lipids, i.e., TAGs and sterol esters, and contain a surrounding layer of phospholipids [28]. In addition, DAGs are incorporated into lipid droplets but have also been implicated in affecting the insulin signaling cascade in insulin resistance [29]. Ceramides, another class of intermediates, are also associated with lower insulin sensitivity [30]. With our lipidomic approach, we demonstrate how individual lipid classes are changing over 24 h. Of all the lipids we measured in human skeletal muscle, we found that 13% exhibit a diurnal profile. Remarkably, more than half of the rhythmic lipids were DAGs. These DAGs showed similar rhythmicity, all dropped at 1 PM and reached their peak level at 4 AM. This rhythmicity of DAGs is interesting, as DAG levels have been associated with reduced insulin sensitivity, and it has been described before that insulin sensitivity is lower in the evening compared to the morning [31]. The TAGs and glycerophospholipids that we found to be rhythmic followed the same diurnal profile. Deviations from this average diurnal profile in these clusters were typically associated with the fatty acid chain length and the number of double bonds. The two other lipid clusters, i.e., sphingolipids and sterol lipids, had a different diurnal profile, they reached peak levels at 6 PM, and the variation in their diurnal profiles was not affected by fatty acid chain length nor absence or presence of double bonds.

Skeletal muscle gene expression studies revealed that the majority of circadian regulated metabolic genes are those involved in lipid metabolism [32]. Indeed, in a recent circadian lipidomic study, in human skeletal muscle, about 19% of the detected lipids appeared to be rhythmic [10]. This percentage is similar to what we found in the current study, but there are still important differences between the study protocols. We have chosen to study 24 h rhythmicity under normal living conditions, including periods of activity and the consumption of three meals per day. We have chosen to do so, as this may reveal how the rhythmicity of skeletal muscle lipids may be linked to physiological variations in muscle metabolism. In contrast, Loizides-Mangold et al. investigated the true circadian rhythmicity of muscle lipids by limiting physical activity and by providing hourly, small snacks [10]. Another difference between the two studies is the lipidomic platform that was used. For instance, DAGs were not included in the measurements of Loizides-Mangold et al. The percentage of rhythmic phospholipids was similar, yet, the rhythmicity pattern of some of the individual lipid classes was distinct. In our study, ceramides peaked at the beginning of the day, while in the study by Loizides-Mangold et al. they peaked at night. Likewise, an association between the profile of rhythmic lipids and chain length was observed. This coincided with gene expression profiles of genes involved in fatty acid elongation and beta-oxidation of (very) long-chain fatty acids [10]. A previous circadian lipidomic study in human plasma showed that 13% of the detected lipidome is rhythmic [9]. The biggest cluster found to be rhythmic in plasma was glycerolipids, predominantly TAGs and DAGs. Similar to our results in muscles, these rhythmic glycerolipids also peaked at late night or early morning while sphingolipids peaked in the afternoon or evening. Moreover, LC-PUFAs often had distinct diurnal profiles [10]. In our study, some glycerophospholipids with LC-PUFAs (e.g., BMP42:9, Figure 4C) showed a distinct profile that was different from the average profile of the lipid class. The total pool of LC-PUFAs such as those containing ≥C20 comes from a combination of absorption from diet, activity of elongases (ELOVLs), and fatty acid oxidation. In the skeletal muscle, the gene expressions of some of these enzymes that specifically handle LC-PUFAs, such as fatty acid elongase 5 and long-chain fatty acyl-CoA synthase 4/5, are under circadian regulation [10].

DAGs are bioactive metabolites that are present as intermediates during the esterification of fatty acids to TAGs, mediated by diacylglycerol acyltransferase (DGAT), or during the hydrolysis of TAGs by lipases such as the hormone-sensitive lipase (HSL). DAGs can also be synthesized from phosphatidic acid (PA). The conversion of PA into DAG is catalyzed by phosphatidate phosphatase-1 activity present in lipin proteins. We found many DAGs but not PA species to be rhythmic, suggesting a diurnal regulation in either the TAG hydrolysis or fatty acid esterification. Indeed, genes involved in DAG biosynthesis (*Lpin1/2, Pnpla3*), TAG hydrolysis (*Atgl* and *Hsl*), and conversion to TAG (*Dgat2*) exhibit circadian regulation in mice [33,34]. Importantly, a recent 24-hour transcriptomic analysis of skeletal muscle biopsies in healthy individuals revealed that the expressions of lipid metabolism genes, such as PPARD, are rhythmic [35]. Moreover, it was reported that the expression of *Lpin1* is blunted after disrupting the molecular clock in human primary myotubes, suggesting clock-control of this enzyme [35].

In a recent study in human muscle [36], about half of all detected DAGs were located in lipid droplets and another part was located at the cell membrane. Ceramides, however, were mostly membrane-bound. Here, we found that oscillating ceramides followed a rhythmic profile that was opposite of the DAG profile, high in the afternoon and low at night. The importance of spatial regulation has previously been shown when the mitochondrial and nuclear lipidome of the murine liver were separated to reveal distinct regulation of oscillating lipids [37]. Since DAGs and ceramides are both implicated in the development of insulin resistance, lipid droplets might help to prevent detrimental effects sequestering such lipids [38]. However, further studies are needed to investigate the specific content of lipid droplets during a day-night cycle.

### 4.3 Conclusions

Taken together, in the current study, we have identified rhythmicity in lipid droplets and the lipidome of human skeletal muscle under real-life-mimicking free-living conditions. Accumulation of lipotoxic intermediates has been associated with metabolic disorders such as diabetes and nonalcoholic fatty liver disease. Future research is required to establish the regulation of diurnal regulation of lipid droplet content in the initiation and progression of said metabolic disorders.

## Appendix A Supplementary data

Multimedia component 1 Multimedia component 1

Multimedia component 2 Study design of the two-day laboratory protocol. Subjects remained under free-living conditions in the metabolic research lab. M denotes the timepoints of biopsies. Three meals were served according to the individual caloric requirements. B, breakfast; L, lunch; D, dinner. Grey area represents the sleeping period (11 PM–7 AM). Multimedia component 2

Multimedia component 3 Separation of DAGs based on chain length and saturation. DAGs are clustered either (A) by chain length or (B) by saturation. Amplitude (the difference between peak and trough) increases with (C) increasing chain length and (D) increasing saturation. Grey area represents sleeping periods (11 PM–7 AM). ∗*P* ≤ 0.05 for the difference between clusters (ANOVA). Multimedia component 3

Multimedia component 4 Lipid droplet morphology and overall IMCL content of the combined type I and type II fibers. The combined fiber type represents the summary of type I and type II fibers. (A) Total IMCL is depicted as the product of lipid droplet size (μm^2^) and number (#/μm^2^). Other figures show (B) lipid droplet size and (C) lipid droplet number. Grey area represents sleeping periods (11 PM–7 AM). ∗ *P* ≤ 0.05 for the effect of time. Multimedia component 4

Multimedia component 5 Individual data from each participant for lipid droplet size and number. (A) Lipid droplet size and (B) number of oxidative type I fibers. (C) Lipid droplet size and (E) number of glycolytic type II fibers. Grey area represents sleeping periods (11 PM–7 AM). Some participants could not be quantified at all time points. ∗ *P* ≤ 0.05 for the effect of time. Multimedia component 5

Multimedia component 6 Individual lipid species from the main lipid clusters. (A) Diradylglycerols, (B) triradylglycerols, (C) glycerophospholipids, (D) sphingolipids, and (E) sterols. Each line represents one lipid species and is the average of all participants. Grey area represents sleeping periods (11 PM–7 AM). Multimedia component 6
