# Supplementary figures and images for: Skeletal muscle in healthy humans exhibits a day-night rhythm in lipid metabolism
Source: Mol Metab. 2020 Apr 6;37:100989. doi: 10.1016/j.molmet.2020.100989 (PMC7217992; doi:10.1016/j.molmet.2020.100989)

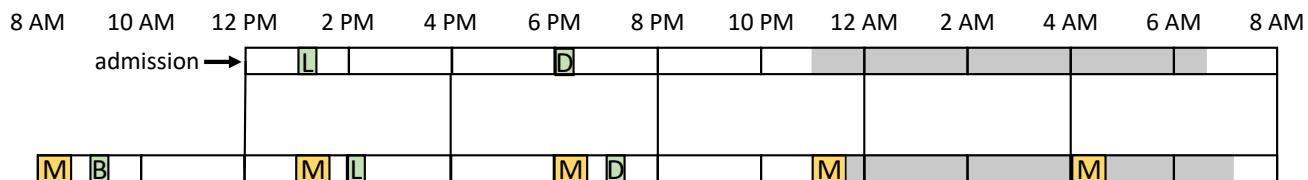

scheduled sleep

■ B = Breakfast, L = Lunch, D = Dinner

■ M Muscle Biopsy

Supplement: Multimedia component 2 — Study design of the two-day laboratory protocol. Subjects remained under free-living conditions in the metabolic research lab. M denotes the timepoints of biopsies. Three meals were served according to the individual caloric requirements. B, breakfast; L, lunch; D, dinner. Grey area represents the sleeping period (11 PM–7 AM). [file mmc2.pdf]

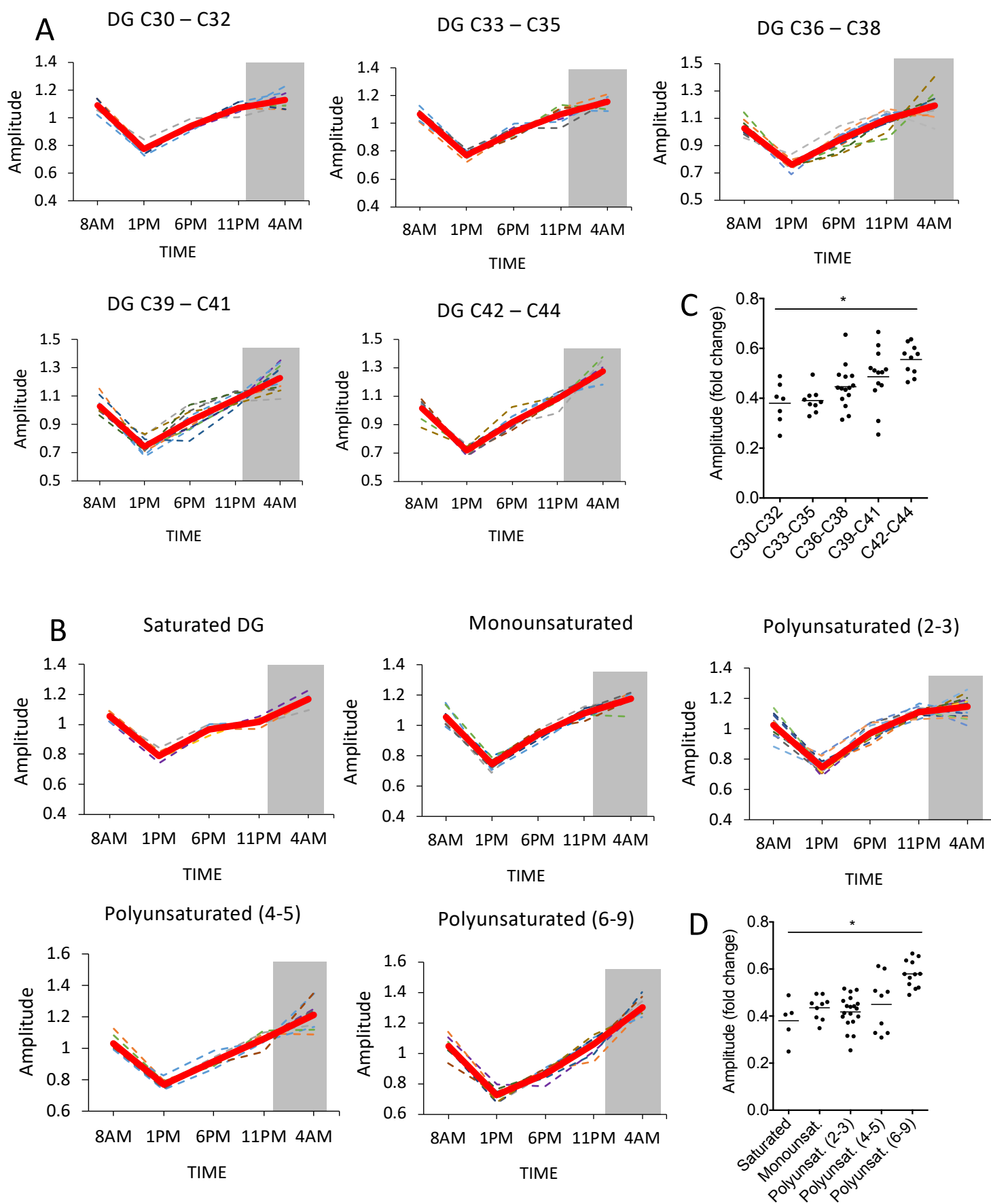

Supplement: Multimedia component 3 — Separation of DAGs based on chain length and saturation. DAGs are clustered either (A) by chain length or (B) by saturation. Amplitude (the difference between peak and trough) increases with (C) increasing chain length and (D) increasing saturation. Grey area represents sleeping periods (11 PM–7 AM). ∗P ≤ 0.05 for the difference between clusters (ANOVA). [file mmc3.pdf]

A

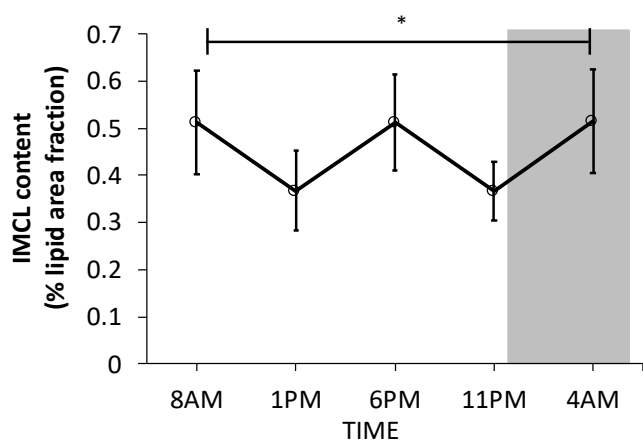

B

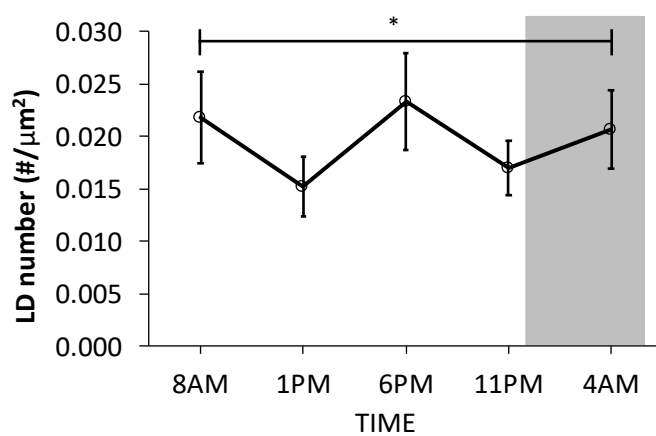

D

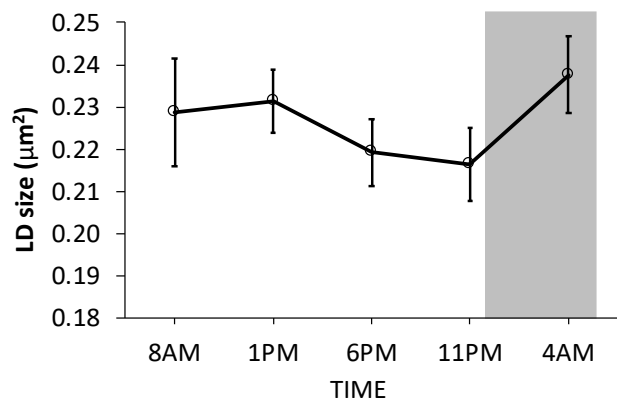

Supplement: Multimedia component 4 — Lipid droplet morphology and overall IMCL content of the combined type I and type II fibers. The combined fiber type represents the summary of type I and type II fibers. (A) Total IMCL is depicted as the product of lipid droplet size (μm2) and number (#/μm2). Other figures show (B) lipid droplet size and (C) lipid droplet number. Grey area represents sleeping periods (11 PM–7 AM). ∗ P ≤ 0.05 for the effect of time. [file mmc4.pdf]

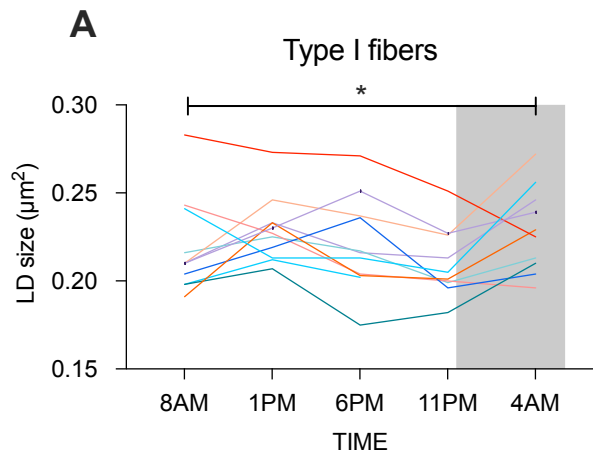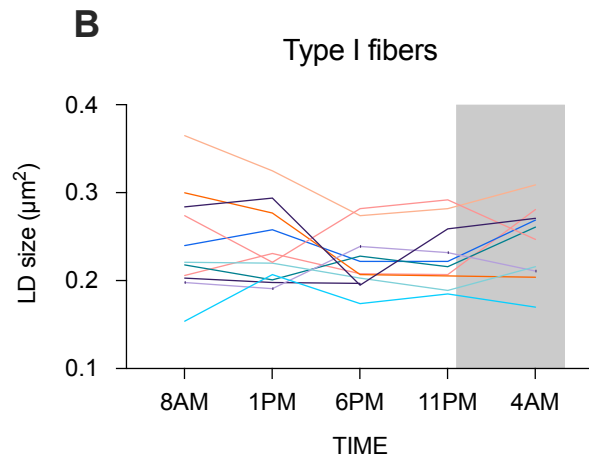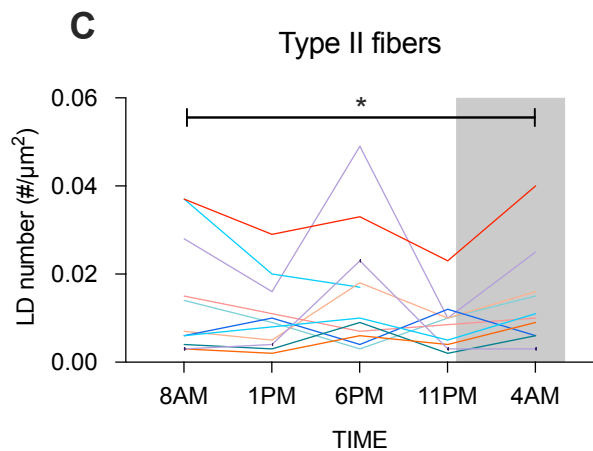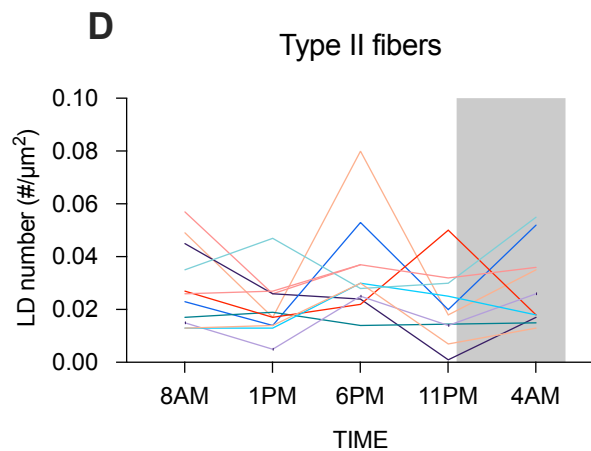

Supplement: Multimedia component 5 — Individual data from each participant for lipid droplet size and number. (A) Lipid droplet size and (B) number of oxidative type I fibers. (C) Lipid droplet size and (E) number of glycolytic type II fibers. Grey area represents sleeping periods (11 PM–7 AM). Some participants could not be quantified at all time points. ∗ P ≤ 0.05 for the effect of time. [file mmc5.pdf]

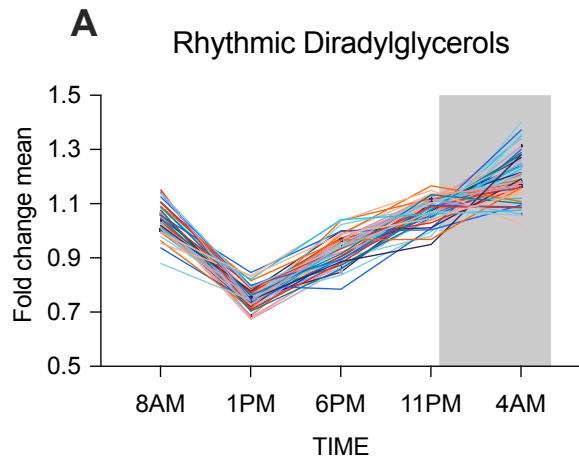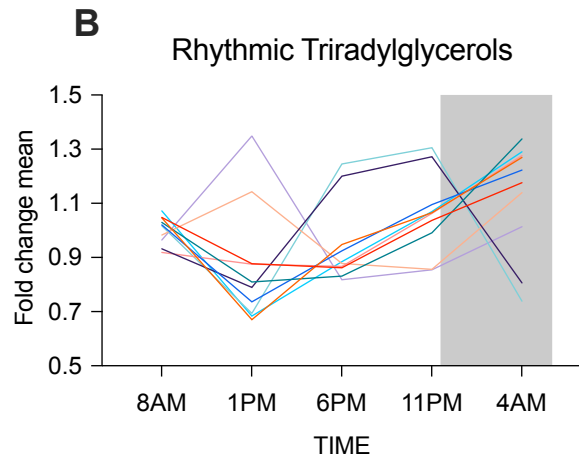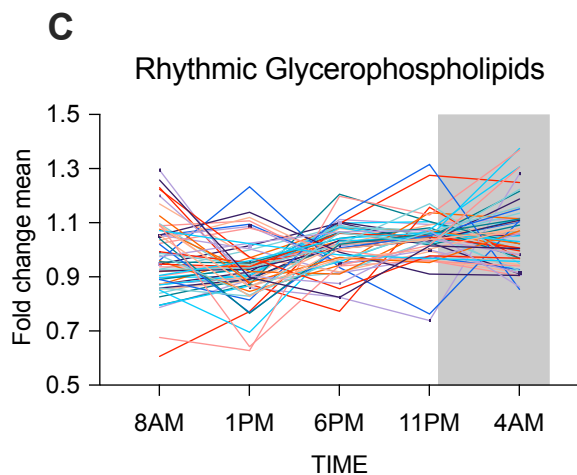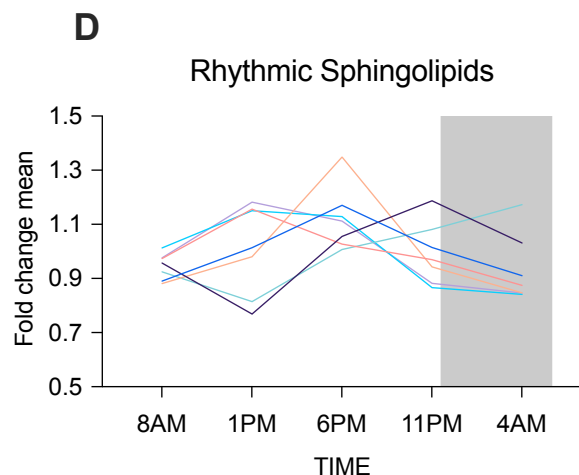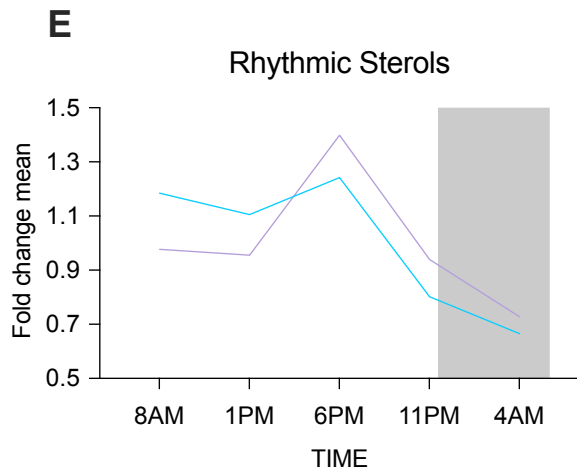

Supplement: Multimedia component 6 — Individual lipid species from the main lipid clusters. (A) Diradylglycerols, (B) triradylglycerols, (C) glycerophospholipids, (D) sphingolipids, and (E) sterols. Each line represents one lipid species and is the average of all participants. Grey area represents sleeping periods (11 PM–7 AM). [file mmc6.pdf]
